# Targeting Endocannabinoid Signaling in the Lateral Habenula as an Intervention to Prevent Mental Illnesses Following Early Life Stress: A Perspective

**DOI:** 10.3389/fnsyn.2021.689518

**Published:** 2021-05-28

**Authors:** Ryan D. Shepard, Fereshteh S. Nugent

**Affiliations:** Department of Pharmacology, F. Edward Hebert School of Medicine, Uniformed Services University of the Health Sciences, Bethesda, MD, United States

**Keywords:** CB1R, early life stress, endocannabinoids, FAAH, lateral habenula, LHb, MAGL

## Abstract

Adverse events and childhood trauma increase the susceptibility towards developing psychiatric disorders (substance use disorder, anxiety, depression, etc.) in adulthood. Although there are treatment strategies that have utility in combating these psychiatric disorders, little attention is placed on how to therapeutically intervene in children exposed to early life stress (ELS) to prevent the development of later psychopathology. The lateral habenula (LHb) has been a topic of extensive investigation in mental health disorders due to its prominent role in emotion and mood regulation through modulation of brain reward and motivational neural circuits. Importantly, rodent models of ELS have been shown to promote LHb dysfunction. Moreover, one of the potential mechanisms contributing to LHb neuronal and synaptic dysfunction involves endocannabinoid (eCB) signaling, which has been observed to critically regulate emotion/mood and motivation. Many pre-clinical studies targeting eCB signaling suggest that this neuromodulatory system could be exploited as an intervention therapy to halt maladaptive processes that promote dysfunction in reward and motivational neural circuits involving the LHb. In this perspective article, we report what is currently known about the role of eCB signaling in LHb function and discuss our opinions on new research directions to determine whether the eCB system is a potentially attractive therapeutic intervention for the prevention and/or treatment of ELS-associated psychiatric illnesses.

## Introduction

Early life stress (ELS) and childhood trauma have been recognized as critical risk factors that increase the probability of developing psychiatric disorders in both clinical observations and animal models (Heim and Nemeroff, 2002; Taylor, 2010; Targum and Nemeroff, 2019). Given our incomplete understanding of the neurobiological underpinnings and mental outcomes following ELS, as well as the wide array of maladaptive alterations in neural circuits induced by ELS, the development of efficacious interventional therapy has been hampered. At the time of writing this perspective, the coronavirus (COVID-19) pandemic has fundamentally changed societal norms with detrimental economic impact leading to increased psychological and physical stress resulting from a multitude of factors such as unemployment, social isolation and homelessness (Salari et al., 2020). Importantly, one of the deleterious effects of the COVID-19 pandemic has been the closure of schools resulting in social isolation of children from their peers and disproportional access to educational resources, which are especially lacking in minority communities and children with behavioral/cognitive deficits (Wong et al., 2020). In addition, there have been concerns of possible increased child maltreatment and neglect, as well as food and housing insecurity due to pandemic shutdowns across the globe (Humphreys et al., 2020). As such, growing epidemiological evidence suggests that adolescents living through the pandemic are potentially at a greater risk for depression and anxiety (Gotlib et al., 2020; Guessoum et al., 2020). Therefore, the issue of the consequences of ELS on the future well-being of children, now more than ever, are at the forefront of public health and further necessitates a complete understanding of the etiology of behavioral disorders associated with ELS and the identification of potential novel interventional therapeutic targets.

In conjunction with efforts aimed at increasing our knowledge of the molecular and cellular alterations that perturb the central nervous system (CNS) function in mental health disorders, identifying critical brain regions and neural circuits has remained of high importance. Stress-induced dysfunction in a number of brain regions following ELS has been linked to stress-related disorders including the amygdala (Malter Cohen et al., 2013), prefrontal cortex (Herzberg and Gunnar, 2020), and ventral tegmental area (VTA; Shepard and Nugent, 2020). Although these structures have been extensively studied, evidence now suggests that the lateral habenula (LHb), an evolutionarily conserved epithalamic structure, is a critical brain region that is involved in depression, substance use disorder, anxiety, and sleep disorders (Hu et al., 2020). Earlier studies from our lab and others have demonstrated that rodent models of ELS promote LHb dysfunction through increased LHb neuronal excitability and altered synaptic transmission (Tchenio et al., 2017; Authement et al., 2018; Langlois et al., 2020; Simmons et al., 2020). Moreover, our longitudinal studies have demonstrated that these ELS-induced alterations are potentially long–lasting and span across multiple stages of development (Shepard et al., 2018b; Langlois et al., 2020).

## Materials and Methods

All experiments were conducted with approval from the Uniformed Services University (USU) Institutional Animal Care and Use Committee (IACUC) and guidelines from the National Institutes of Health (NIH) for the care and use of laboratory animals.

### Maternal Deprivation and LHb Microdissection

Maternal deprivation (MD) was carried out as described previously (Authement et al., 2018; Shepard et al., 2018a,b, 2020; Simmons et al., 2020). At P9, male pups (Charles River) were isolated from the dam as a group in a separate room to avoid localization from other animals in the main housing area. MD pups were kept together and isolated from the dam for a total of 24 h before being returned to the home cage with the dam and remaining pups serving as the control group (non-maternally deprived, non-MD rats). Rats were kept with the dam until P28 where they were weaned into dyads based on stress conditions (non-MD vs. MD). Between postnatal days 70–80, adult male rats were anesthetized with isoflurane and decapitated by guillotine. Brains were quickly dissected in ice-cold artificial cerebrospinal fluid (ACSF) containing (in mM): 126 NaCl, 21.4 NaHCO_3_, 2.5 KCl, 1.2 NaH_2_PO_4_, 2.4 CaCl_2_, 1.00 MgSO_4_, 11.1 glucose, 0.4 ascorbic acid, saturated with 95% O_2_–5% CO_2_. Sagittal brain sections were taken at 300 μm and the LHb was grossly dissected from each as done previously (Authement et al., 2018). LHb was visualized by the slice containing both the stria medullaris and the fasciculus retroflexus.

### Western Blot

WBs on LHb tissues were conducted as described previously (Authement et al., 2018). Twenty microgram of LHb protein lysates were loaded into precast polyacrylamide gels and separated by electrophoresis. Following transfer to nitrocellulose membranes, antibodies targeted against MAGL (1:500, Millipore ABN1000), FAAH (1:500, Abcam ab54615), GAPDH (1:1,000, Abcam ab9485) and vinculin (1:1,000, Abcam ab129002). HRP-linked secondary antibodies targeting either rabbit (1:2,000, Cell Signaling) or mouse (1:2,000, Cell Signaling) were used. Data was analyzed using ImageJ. MAGL and FAAH expression levels were normalized to the levels of either GAPDH or vinculin. Summary data is reported as a fold change and is normalized to the non-MD group.

### Statistical Analysis

Values for WB experiments are presented as means ± SEM. Statistical significance was determined using unpaired Student’s *t*-test and was set at a level of *p* < 0.05. Due to a smaller sample size, the Shapiro-Wilk test was used to assess normality. Statistical analyses were performed using GraphPad Prism.

### The Lateral Habenula: a Lynchpin for the Development of Psychiatric Disorders?

The habenula (both medial and lateral divisions) together with the stria medularis and pineal gland (Roman et al., 2020) make up the epithalamus which is conserved from humans down to teleosts indicating a critical evolutionary role (Sutherland, 1982). Specifically, the LHb functions as an “anti-reward” structure which is activated in response to aversion and negative reward (Hikosaka, 2010). Serving as a critical hub for incoming afferents projecting from forebrain and limbic regions (Hikosaka et al., 2008; Hu et al., 2020; Roman et al., 2020), the primarily glutamatergic pathways from the LHb suppress the release of monoamines such as dopamine (DA) and serotonin from the VTA (Ji and Shepard, 2007; Matsumoto and Hikosaka, 2007; Hong et al., 2011) and dorsal raphe nucleus (DRN; Pasquier et al., 1976; Varga et al., 2003), respectively, through their direct and indirect (GABAergic neurons of the rostromedial tegmental area, RMTg) projections. However, recent evidence suggests the existence of possible GABAergic interneurons in the LHb that could also provide local inhibitory signaling (Zhang et al., 2018; Flanigan et al., 2020; Webster et al., 2020; Li et al., 2021). It is important to note that LHb glutamatergic neurons are not homogenous and differ with respect to their physiological firing patterns, the expression of a variety of neuropeptidergic receptors, their downstream projections, and the inputs that they receive (Wallace et al., 2020). Studies have suggested that dysregulation of LHb function is involved in the pathophysiology of a variety of mental disorders (Lecca et al., 2014; Hu et al., 2020). Most notably, LHb hyperactivity has been consistently found in both clinical and animal models of depression (Proulx et al., 2014; Browne et al., 2018; Nuno-Perez et al., 2018; Yang et al., 2018; Cerniauskas et al., 2019; Gold and Kadriu, 2019). While LHb hyperactivity associated with depressive phenotypes have been found to occur uniformly across all LHb neuronal subpopulations (Li et al., 2013; Authement et al., 2018; Shepard et al., 2018b; Yang et al., 2018; Simmons et al., 2020), some studies suggest projection- and input-specific LHb subcircuit dysfunction in depression (Li et al., 2011; Cerniauskas et al., 2019). For example, the enhanced release of glutamate at glutamatergic synapses onto VTA-projecting LHb neurons correlates with learned helplessness behaviors in rats (Li et al., 2011) suggesting that depression-related hyperactivity in specific LHb subpopulations may arise from synaptic changes at distinct synaptic inputs to LHb subpopulations. Additionally, the entopeduncular (EP) nucleus (rodent homolog to the internal segment globus pallidus) co-releases glutamate/GABA to mediate aversion and “anti-reward” signaling. Antidepressants can alter changes in presynaptic glutamate/GABA release and thereby modulate LHb neuronal excitability (Shabel et al., 2012, 2014; Cerniauskas et al., 2019; Wallace et al., 2020). Consistently, chronic stress in mice increases the activity of VTA- but not DRN-projecting LHb neurons through enhanced presynaptic glutamate release from the EP, which underlies stress-induced increases in passive coping and reduced motivation, but not anxiety or anhedonia (Cerniauskas et al., 2019). Given that DA dysfunction is associated with ELS (Pruessner et al., 2004), our lab first focused on the effects of ELS on VTA DA function using an ELS model of MD in rats (Ellenbroek et al., 2005) in which rat pups are subjected to a single 24-h maternal separation (MS) from the dam. We demonstrated that MD induced VTA DA dysfunction through induction of GABAergic metaplasticity involving A-kinase anchoring scaffolding protein (AKAP150, also referred to AKAP79—the human equivalent to rodent AKAP150) signaling and histone deacetylases (HDACs). We showed that MD in juvenile rats selectively induces long-term depression (LTD) and shifts spike-timing-dependent plasticity (STDP) toward LTD at GABAergic synapses onto VTA DA neurons. This MD-induced metaplasticity involved epigenetic modifications to AKAP150 signaling that included an increase in HDAC2 and decreased histone acetylation which was reversible with HDAC inhibition (Authement et al., 2015; Shepard et al., 2018a, 2020; Shepard and Nugent, 2020). Taken together, this body of work demonstrates that MD alters GABAergic synaptic strength onto VTA DA neurons which potentially contributes to DA dysfunction in psychiatric disorders stemming from ELS.

Considering that the LHb regulates VTA DA signaling, we extended our studies to determine whether MD also perturbs LHb function. Studies from our lab using MD and others using maternal separation (MS; Tchenio et al., 2017) or the limited bedding and nesting models of ELS (Bolton et al., 2018) demonstrated that ELS can promote LHb hyperactivity (Authement et al., 2018; Shepard et al., 2018b; Simmons et al., 2020). Mechanistically, we have shown that LHb neurons in adolescent rats that underwent MD stress are hyperexcitable partly due to a shift in synaptic excitation and inhibition (E/I) balance towards excitation, as well as downregulation of small conductance (SK2) potassium channels and increased protein kinase (PKA) activity, resulting in induction of an intrinsic plasticity in LHb neurons. On the other hand, MS stress in mice decreases postsynaptic GABA_B_R signaling arising from entopeduncular nucleus GABAergic inputs which then contributes to MS-induced LHb hyperexcitability in adult mice (Tchenio et al., 2017). Given the epidemiological evidence for the increased predisposition towards developing psychiatric conditions of children subjected to early trauma, we also aimed to determine whether MD-induced LHb hyperactivity persists throughout adolescence into adulthood. Indeed, we have demonstrated persistent maladaptive alterations in LHb neuronal and synaptic function by MD from early adolescence (Authement et al., 2018; Shepard et al., 2018b; Simmons et al., 2020) into adulthood (Langlois et al., 2020). We also found that one of the possible mechanisms contributing to synaptic dysfunction in the LHb during early adolescence may be related to the potential impairment of eCB signaling (Authement et al., 2018) which we hypothesize partly contributes to the long-lasting ELS-induced LHb hyperexcitability.

### eCB Signaling: a Key Player in Synaptic Regulation

Unlike neurotransmitters and neuropeptides, eCBs are lipids that are synthesized on-demand in an activity-dependent manner. The two major constituent eCB signaling messengers are N-arachidonoylethanolamine (anandamide; AEA) and 2-arachidonoylglycerol (2-AG) which primarily act on cannabinoid receptors 1 and 2 (CB1R and CB2R) as retrograde signals, although they can also act on transient receptor potential vanilloid receptor type 1 (TRPV1) as non-retrograde signals (Castillo et al., 2012). There is a great deal of evidence to support that both AEA and 2-AG are synthesized by enzymes such as *N*-acylphosphatidylethanolamine-phospholipase D (NAPE-PLD) and diacylglycerol lipase (DAGL), respectively, that are localized to postsynaptic neurons (Katona et al., 2006; Yoshida et al., 2006; Yoshino et al., 2011). Interestingly, eCB degradation enzymes are segregated spatially in that fatty acid amide hydrolase (FAAH, the main degradative enzyme for AEA) is principally found postsynaptically and monoacylglycerol lipase (MAGL, the enzyme responsible for 2-AG degradation) is found presynaptically. Given these differences in spatial organization, it is reasonable to assume that these enzymatic synthesis/degradation pathways can be distinctly modulated with cell type-and brain region-specificity to regulate neuronal activity, synaptic strength (balance and/or coordination of excitatory to inhibitory inputs), and plasticity. Although there are a wide variety of receptors that eCBs can target independently of CB1Rs/CB2Rs, such as TRPV channels and other orphan receptors (Cristino et al., 2020), we will be discussing our perspective on eCB signaling within the context of CB1R engagement due to its high neuronal expression (Qureshi et al., 1998) and robust role in synaptic plasticity (Gerdeman and Lovinger, 2003; Castillo et al., 2012). We recommend the following in-depth reviews for the role of eCB signaling in synaptic function and plasticity (Castillo et al., 2012; Augustin and Lovinger, 2018; Zou and Kumar, 2018; Cristino et al., 2020).

CB1Rs are G_i/o_-protein-coupled receptors (GPCRs) which once activated decrease the release of neurotransmitter presynaptically through a variety of mechanisms including decreased cyclic adenosine monophosphate (cAMP), membrane hyperpolarization through activation of potassium channels and decreased vesicular release of neurotransmitter *via* inhibition of voltage-gated calcium channels (VGCCs; Cristino et al., 2020). Although CB1Rs are primarily considered to be presynaptically localized, studies have also identified that CB1Rs can also function as autoreceptors when postsynaptically localized (Bacci et al., 2004). Lastly, others have identified that CB1Rs can also localize to glial cells, such as astrocytes (Navarrete and Araque, 2008). Moreover, depending on the localization of CB1Rs at distinct presynaptic inputs, eCBs are poised to differentially affect the strength of synaptic transmission in a cell-type and circuit-dependent manner. Importantly, eCBs are developmentally regulated and their levels fluctuate during critical developmental windows (Meyer et al., 2018). Not surprisingly, disease-based alterations in the neural function and behaviors involving eCB signaling have been implicated, making the eCB system a potential therapeutic target. Given the complexity of the eCB system that also includes several eCB-related mediators, their enzymes, and their molecular targets, the classical eCB signaling has been expanded to an "-ome"; the endocannabidiome (Cristino et al., 2020).

### ELS Potentially Impairs eCB Signaling in the LHb

Studies on LHb physiology have demonstrated that eCB signaling profoundly controls LHb synaptic plasticity, neuronal activity, and associated behaviors (Valentinova and Mameli, 2016; Park et al., 2017; Authement et al., 2018; Berger et al., 2018). Potential alterations in eCB-mediated regulation of LHb neurons could contribute to LHb dysfunction associated with anhedonia, as well as motivational and social deficits in depression and other stress-related disorders.

In fact, CB1Rs are shown to be highly expressed in the LHb, specifically in presynaptic axon terminals, postsynaptic dendrites and glia (Berger et al., 2018). Intra-LHb manipulations of CB1Rs, has been shown to bi-directionally affect stress coping strategies where upon LHb CB1R blockade, rats display a more proactive behavioral style over avoidance (i.e., rats are more likely to explore and interact with a novel conspecific) whereas LHb CB1R activation increases avoidant behaviors (Berger et al., 2018). These data suggest that LHb eCB signaling has profound effects on motivational and social behaviors which could be mediated by eCB-mediated synaptic modifications in the LHb. The two forms of synaptic plasticity (long-term potentiation, LTP, and LTD) are both subject to eCB modulation (Castillo et al., 2012), although eCBs mostly depress synaptic transmission (through a decrease in presynaptic neurotransmitter release), inhibit LTP and promote the induction of a presynaptic LTD at both glutamatergic and GABAergic synapses (Castillo et al., 2012). In response to a traditional LTD protocol (low frequency stimulation), or upon activation of group I metabotropic glutamate receptor (mGluR I), LHb neurons can express an eCB-mediated presynaptic LTD at glutamatergic synapses (Valentinova and Mameli, 2016; Park et al., 2017). Importantly, a strong stressor (delivering unpredictable tail shocks while rats are restrained) is sufficient to block eCB-mediated glutamatergic LTD in LHb neurons, potentially enhancing the excitatory drive onto LHb neurons following this acute stress. The stress-induced impairment of eCB-mediated LTD is recovered by inhibition of CamKIIa (an important LHb molecular substrate in behavioral depression; Park et al., 2017). Interestingly, mGluR activation also triggers LTD at GABAergic synapses through PKC-dependent reduction of β2-containing GABA_A_Rs in LHb neurons suggesting that LHb mGluR signaling bidirectionally regulates LHb neuronal outputs through induction of mGluR-dependent synaptic plasticity that could mediate opposing motivational behaviors (Valentinova and Mameli, 2016).

We also found that activation and inhibition of CB1Rs decreases and increases the probability of presynaptic GABA release in the LHb, respectively, suggesting a possible presence of a tonic eCB signaling and persistent CB1 receptor activation in the regulation of GABAergic transmission in the LHb (Authement et al., 2018). Importantly, we have shown that activation of corticotropin-releasing factor (CRF)-CRFR1-PKA signaling increases LHb excitability through selective suppression of presynaptic GABA release onto LHb neurons through retrograde eCB-CB1R signaling, as well as increases in intrinsic excitability of LHb neurons through PKA-dependent reduction of SK2 channels. More importantly, we observed blunted responses of LHb neurons to the excitatory effects of acute CRF signaling in LHb neurons due to MD-induced increases in PKA activity, possibly downregulating SK2 channels in LHb neurons (Authement et al., 2018).

**Figure 1:**
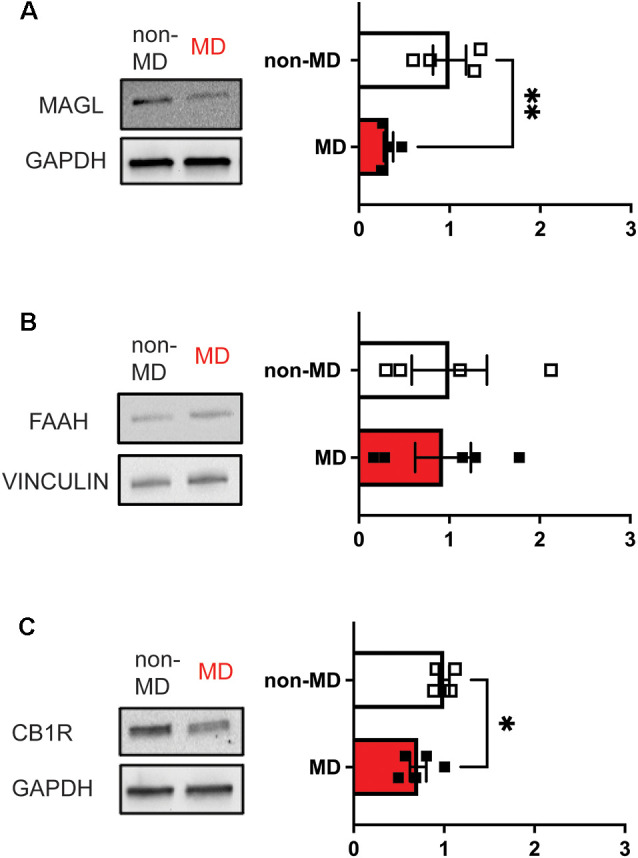
Maternal deprivation (MD) decreases the abundance of both MAGL and CB1R in LHb whole protein lysates, but not FAAH in adulthood (Postnatal days 70–80) in response to ELS at Postnatal day 9. Bar graphs represent fold changes in the abundance of protein with respect to non-MD rats. **(A)** MD decreases total MAGL abundance (non-MD, *n* = 4; MD, *n* = 4; *t*_(6)_ = 3.543). **(B)** MD does not significantly affect FAAH abundance (non-MD, *n* = 4; MD, *n* = 5; *t*_(7)_ = 0.1360). **(C)** MD decreases CB1R abundance (non-MD, *n* = 4; MD, *n* = 5; *t*_(7)_ = 2.522). *p < 0.05, **p < 0.01 by unpaired *t*-test.

Our longitudinal studies on the effects of MD on LHb function demonstrated the persistence of MD-induced LHb hyperexcitability during development (Shepard et al., 2018b; Langlois et al., 2020). Interestingly, we have also observed potentially long–lasting elevations in eCB-2AG levels in the LHb as we found decreased MAGL (2-AG degrading enzyme) protein levels, as well as decreases in CB1R expression in the adult rat LHb (Figure 1). This is in line with the studies from knock-out (KO) mice where either deletion of MAGL or chronic antagonism of MAGL can decrease CB1R expression in the CNS (Chanda et al., 2010; Schlosburg et al., 2014). Consistently, chronic unpredictable stress has been shown to alter eCB degradation and is associated with less CB1R binding (Berger et al., 2018). Similarly, a recent study demonstrated that exposure to alcohol in rats decreases CB1R expression, but in contrast, increases MAGL with no change in FAAH. Interestingly, this study also found that activation of eCB signaling within the LHb is an analgesic and could reduce ethanol intake (Fu et al., 2021). Additionally, KO of MAGL can promote anxiety- and pro-depressive behaviors (Imperatore et al., 2015) providing additional evidence for a critical role of eCB modulation of neural circuits that regulate stress-related behaviors. Given that adult MD rats show an imbalance of excitation and inhibition towards excitation with LHb hyperactivity (Shepard et al., 2018b; Langlois et al., 2020), we assume that this could also contribute to an elevation in eCB 2-AG production. Although we show the interaction between CRF neuromodulatory systems with eCB signaling within the adolescent rat LHb following MD, whether MD-induced dysregulation of CRF signaling persists into adulthood and affects eCB-mediated regulation of LHb activity is unclear. Given that MD-induced CRF dysregulation seems to promote intrinsic LHb excitability and that eCB-CB1R signaling decreases both presynaptic glutamate and GABA release and mediates glutamatergic LTD in LHb, we predict that MD may decrease 2-AG degradation by MD-induced decreases in presynaptic MAGL specific to presynaptic GABAergic inputs to the LHb while selective MD-induced downregulation of CB1Rs in presynaptic glutamatergic terminals within the LHb could decrease eCB-mediated suppression of glutamate release as well as inhibit eCB-mediated LTD in LHb neurons. Interestingly, a recent study demonstrated that most cannabinoids suppress the reinforcing effects of optogenetic VTA DA neuron self-stimulation in mice, suggesting that cannabinoid receptor activation, in general, attenuates VTA DA reward or could exert aversive effects. This study also shows that VTA GABA and glutamate neurons express CB1Rs while VTA DA neurons express CB2Rs (Humburg et al., 2021). Given that VTA GABA neurons also provide inhibitory GABAergic input to LHb, it is possible that ELS-induced decreases in MAGL expression and persistent increases in eCB 2AG-CB1R-mediated suppression of VTA GABAergic input to the LHb promotes ELS-associated LHb hyperactivity. However, a technical limitation of our molecular data is the lack of cell-type and synapse-specific localizations of the enzyme and receptor which poses the question whether MD-induced downregulation of CB1R and MAGL occurs globally at all excitatory and inhibitory synaptic inputs and LHb neurons projecting to different downstream targets. Our studies show that MD increases the overall activity of LHb neurons and induces behavioral changes including increased immobility in the forced swim test, reduced sucrose preference, and decreased motivation for morphine self-administration (Shepard et al., 2018b; Langlois et al., 2020). Given that distinct LHb subcircuits may mediate specific behavioral phenotypes as shown following chronic stress model of depression in mice (Cerniauskas et al., 2019), future ELS research is necessary to investigate the precise contribution of ELS-induced hyperactivity of VTA-, RMTg- and DRN-projecting LHb neurons as well as eCB-mediated synaptic plasticity at specific synaptic inputs to the LHb in different behavioral deficits following ELS. Moreover, it is worthwhile to examine how different ELS models (MD, MS or LBN) affect stress neuromodulatory systems such as CRF (Authement et al., 2018) and kappa opioid receptor signaling (Simmons et al., 2020) within the LHb with their possible interaction with the eCB system in mediating motivational and anhedonic states associated with these ELS models as cell type and circuit-specific manner.

## Discussion

New advances in understanding neural circuits and the connectome have shaped our understanding of how discrete brain regions and circuits become dysregulated in psychiatric and neurological disorders. Given that eCB signaling plays a critical role in neuronal regulation across the CNS, greater emphasis needs to be placed on the role of eCB signaling with respect to specific inputs and brain structures. Advancements in mouse genetics and Cre-dependent manipulations of neuronal activity (optogenetics and DREADDs) and gene expression allow us to gain greater spatial and temporal control for cell type- and circuit-specific eCB neuromodulation and eCB-mediated behaviors. In addition to using preclinical animal models to study the role of eCB signaling, it is also important to understand whether pharmacological manipulation of eCB signaling in a clinical setting is a viable treatment method. Until recently, one problem with imaging the habenula was due to its small size which makes functional imaging a challenge (Lawson et al., 2013). Recently, using magnetic resonance imaging (MRI) with better resolution has made it possible to functionally image the human habenula (Strotmann et al., 2013, 2014). However, it is still not possible to reliably distinguish between the medial and lateral divisions in humans.

Recent legislative actions have now allowed the use of medical marijuana for the treatment of a variety of conditions which vary across the United States and countries around the world. However, one concern of the use of marijuana and other derivatives as treatment is their potential for abuse, as well as the potential for inducing schizophrenia and other psychiatric disorders (Bostwick, 2012; Chadwick et al., 2013). Therefore, a large number of clinical trials have both been conducted and are currently underway to determine whether targeting CB1R directly or affecting the degradation rate of eCBs through FAAH and MAGL inhibitors can be used in a wide variety of disorders ranging from anxiety to epilepsy (van Egmond et al., 2021); however, there is still a lack of clinical data to determine whether these compounds will be efficacious across the general population. In addition, there is greater concern over whether treatment in pediatrics and adolescents would be safe (Fontanella et al., 2021) considering the critical role of the eCB system in development (Meyer et al., 2018). Therefore, future drug development could potentially focus more so on targeting downstream eCB signaling from CB1Rs.

Advances in proteomic screenings have started to allow the identification of protein-protein interactions of receptor complexes. In fact, many receptors can exist in nature as receptor complexes where accessory proteins or auxiliary subunits can impact receptor trafficking, kinetics, and pharmacology (Maher et al., 2017). Already, these accessory proteins have been identified for VGCCs (Campiglio and Flucher, 2015), AMPARs (Kamalova and Nakagawa, 2021), nAChRs (Boulin et al., 2012), and most recently GABA_A_Rs (Castellano et al., 2020; Han et al., 2020). Indeed, targeting of auxiliary subunits has already succeeded with gabapentin being used for the treatment of epilepsy and pain. Currently, the transmembrane AMPAR regulatory protein (TARP) auxiliary subunit γ8 is in clinical trials for the treatment of pain and epilepsy (Kato et al., 2016; Maher et al., 2017). Targeting receptor complexes offer an exciting opportunity for more precision-based pharmacology and to mitigate off-target effects associated with numerous drugs.

Like other receptors, CB1Rs are expressed ubiquitously in the CNS and targeting CB1R-associated proteins to modify downstream intracellular signaling could perhaps yield novel drug targets for development, such as cannabinoid receptor-interacting protein 1a and 1b (CRIP1a and CRIP1b, respectively). For example, CRIP1a is a CB1R-specific accessory protein (Niehaus et al., 2007) which has been demonstrated to compete with b-arrestin binding which prevents desensitization and internalization of the receptor (Blume et al., 2017). Additionally, CRIP1a can alter CB1R signaling by altering GPCR signaling pathways (Blume et al., 2016) and stopping CB1R-mediated closure of VGCCs (Niehaus et al., 2007). Within this context, it is perhaps possible that our MD-induced decreases in CB1R in adult rat LHb (Figure 1) is due to decreased CRIP1A which allows b-arrestin-mediated endocytosis of CB1Rs; however, this is pure speculation and would need to be verified by both CRIP1a expression, as well as biochemical studies demonstrating the association of both CRIP1a and CB1R in the LHb. Therefore, further research regarding the role of CRIP1a and CRIP1b signaling in a cell type-and circuit-specific manner, in preclinical animal models would provide critical data on the physiological role of these accessory proteins as well as whether they are dysregulated in ELS animal models.

## Conclusion

In summary, given the robust role of the LHb in psychiatric disorders as well as the role of CB1R-mediated regulation of LHb activity, targeting the eCB system for ELS-induced psychiatric disorders is a potential therapeutic option. Although a majority of the clinical data has been conducted in adults, greater observations to the role of eCB signaling in adolescents, as well as possible clinical administration of eCB-targeting compounds could provide data as to their safety and efficacy in younger individuals. Lastly, targeting receptor complexes (Rosenbaum et al., 2020) might be a more precise therapy that can reduce the likelihood of adverse off-target effects and mitigate the aforementioned concerns.

## Data Availability Statement

The original contributions presented in the study are included in the article, further inquiries can be directed to the corresponding author.

## Ethics Statement

The animal study was reviewed and approved by Uniformed Services University IACUC committee.

## Author Contributions

FN and RS designed the experiments and wrote the manuscript. RS performed the experiments, analyzed the data, and prepared the figures. All authors contributed to the article and approved the submitted version.

## Conflict of Interest

The authors declare that the research was conducted in the absence of any commercial or financial relationships that could be construed as a potential conflict of interest.
